# Sex-specific changes in metabolism during the transition from chow to high-fat diet feeding are abolished in response to dieting in C57BL/6J mice

**DOI:** 10.1038/s41366-022-01174-4

**Published:** 2022-07-06

**Authors:** Jennifer Oraha, Ronaldo F. Enriquez, Herbert Herzog, Nicola J. Lee

**Affiliations:** 1 Garvan Institute of Medical Research, Sydney, NSW Australia; 2 School of Clinical Medicine, UNSW Sydney, Sydney, NSW Australia

**Keywords:** Mouse, Obesity, Hypothalamus, Obesity, Obesity

## Abstract

**Background/Objective:**

Female mice are often excluded from diet-induced obesity studies as they are more resistant to the obesifying effects of a high-fat diet (HFD). However, the underlying mechanisms behind this sex disparity may actually have important implications for the development and management of obesity in humans. Therefore, we systematically investigated the immediate sex-specific effects of transitioning to a HFD in C57BL/6J mice as well as monitored whether these effects are altered after sustained HFD feeding and whether sex affects the response to a return to chow, representative of dieting.

**Methods:**

Dual X-ray absorptiometry (DXA) analysis of body composition, indirect calorimetry measurements, and qPCR analysis of hypothalamic and brainstem regions were performed on male and female C57BL/6J mice.

**Results:**

HFD had immediate and dramatic effects in males, increasing fat mass by 58% in the first 3 days. The resistance to the obesifying effect of HFD in females was linked both to an ability to maintain activity levels as well as to an immediate and significantly enhanced reduction in respiratory quotient (RQ), suggesting a greater ability to utilise fat in the diet as a source of fuel. Mechanistically, this sex disparity may be at least partially due to inherent sex differences in the catabolic (POMC/CART) versus anabolic (NPY/AgRP) neurological signalling pathways. Interestingly, the reintroduction of chow following HFD had immediate and consistent responses between the sexes with body composition and most metabolic parameters normalised within 3 days. However, both sexes displayed elevated hypothalamic *Npy* levels reminiscent of starvation. The difference in RQ seen between the sexes on HFD was immediately abolished suggesting similar abilities to burn fat reserves for fuel.

**Conclusions:**

C57BL/6J mice have markedly different sex-specific behavioural and metabolic responses to the introduction as well as the sustained intake of a HFD, but consistent responses to a dieting situation.

## Introduction

Obesity and type 2 diabetes are increasingly common and inter-related chronic conditions with major health and economic impacts affecting vast numbers of people worldwide. The major driver of obesity is excess dietary fat intake through the consumption of low nutrient, energy-dense foods, often coupled with decreased physical activity. Interestingly, the global prevalence of overweight and obesity is higher in women than in men [1]. However, the magnitude of the difference between the sexes does vary greatly by country, often due to complicating behavioural, socio-cultural and socio-economic factors [2]. In developed countries, men actually have a higher rate of being overweight than women and a greater prevalence of type 2 diabetes [3, 4]. Furthermore, in addition to the sex-specific disparities in obesity prevalence, the implications of excess weight gain on health have also been shown to vary by sex [5–8]. It is clear that fundamental sex-specific differences exist in the regulation of metabolic homoeostasis which, along with environmental factors, play a key role in the development of obesity and diabetes [9].

The impact of sex on biological processes is a neglected aspect of biomedical research with the majority of basic and pre-clinical research studies across most disciplines focusing on males only [10, 11]. Whilst the inclusion of females into studies has improved in the last 10 years, studies still predominantly omit sex-based analyses [12]. This is despite the profound influence that sex has on the prevalence, presentation and progression of many diseases and the resultant fact that the exclusion of sex as a factor in studies substantially reduces the applicability of the results for half of the population. Feeding mice a high-fat diet (HFD) is often used as a valuable model in which to study the effects of diet-induced obesity whilst being able to control for variables such as environment and age. Interestingly, most animal models of obesity and type 2 diabetes including diet-induced as well as pharmacologically-induced and genetic models show some degree of sex-specific differences, often with a more severe phenotype in males [13]. However, few studies have systematically investigated the direct effects of sex in these models with many studies solely focusing on male mice. This is true for the commonly used C57BL/6J mouse line in which, upon consumption of a HFD, males develop obesity, hyperinsulinemia, hyperglycaemia and hypertension [14–16]. In contrast, female C57BL/6J mice are more resistant to the obesogenic effects of a HFD [17, 18] for which fact they are often deliberately excluded from studies. However, uncovering the underlying mechanisms of this sex disparity may have important implications for the development and management of obesity in humans.

The metabolic differences between male and female C57BL/6J mice were highlighted in a recent study where both male and female C57BL/6J mice were fed obesifying diets from the young age of 4 weeks for 22 weeks [17]. Significantly different responses were observed after 8 and 16 weeks of HFD feeding between male and female mice in terms of weight gain, food consumption, locomotor activity, energy expenditure and glucose tolerance [17]. However, there is a lack of information regarding the acute sex-specific effects of a HFD in adult mice as well as whether sex-specific differences also exist in situations where dietary fat is reduced, representative of dieting. Therefore, in this study we use male and female adult C57BL/6J mice to examine the sex-specific metabolic differences that occur immediately after transition from chow to a HFD as well as after several weeks of sustained HFD feeding. We also investigate whether there are any sex-specific differences in the metabolic response to transitioning immediately from a HFD back onto chow. We further investigate signalling in the hypothalamus and brainstem in order to begin to determine the underlying mechanisms for these sex-specific effects.

## Methods

### Animals

C57BL/6J mice were obtained from Australian BioResources (Moss Vale, NSW, Australia). All animal experiments were approved by the Garvan Institute/St Vincent’s Hospital Animal Experimentation Ethics Committee and conducted in accordance with relevant guidelines and regulations. Mice were housed under conditions of controlled temperature (22 °C) with a 12 h light, 12 h dark cycle (lights on at 0700 h). Mice had ad libitum access to a standard chow diet (6% fat, 23% protein, 51% carbohydrate, 3.1 kcal/g; catalogue number 27 Mod K1, Gordon’s Speciality Stock Feeds, Yanderra, NSW, Australia) or a HFD (23% fat, 19.4% protein, 46.5% carbohydrate, 4.78 kcal/g; catalogue number SF03-020, Speciality Feeds, WA, Australia). The food quotient (FQ) of the diets was calculated using the following equation to give a value of 0.942 and 0.847 for chow and HFD respectively: (0.835 x % calories from protein) + (1.0 x % calories from carbohydrate) + (0.71 x % calories from fat) [19]. Body weight was monitored weekly.

Eight male and eight female mice at 13 weeks of age were monitored in the Promethion metabolic cages on a chow diet and transitioned to HFD whilst in the system to investigate immediate effects of a transition to HFD. As the Promethion system has only eight cages, this was performed on two separate groups of mice at different times with four males and four females in each run. Dual X-ray absorptiometry (DXA) analyses were performed before Promethion acclimatisation and immediately following the end of the monitoring period. In order to investigate metabolic changes after a prolonged exposure to HFD, a further group of eight male and eight female mice were placed on HFD at 9 weeks of age and body weight was monitored weekly. A control group of seven male and seven female mice were maintained on chow. DXA analyses were performed at 9 weeks of age before HFD started and again prior to acclimatisation in the Promethion system. After 5 weeks on HFD, eight males and eight females on HFD were monitored in the Promethion metabolic cages. Again, this was performed across two separate runs with four males and four females in each run. During these runs, the mice were transitioned back to a chow diet whilst in the Promethion system and their body composition was measured again by DXA immediately following the end of the Promethion monitoring period. However, due to a failure of the DXA machine calibration, one run of four males and four females were unable to have their body composition measured during the diet stage giving an *n* of 4 per sex for the diet group final DXA measurements.

### Metabolic phenotyping of mice

Whole body lean mass, fat mass, bone mineral content (BMC) and bone mineral density (BMD) were measured in mice anesthetised with isoflurane using a dedicated mouse DXA (Lunar Piximus II, GE Medical Systems, Madison WI). Indirect calorimetry measurements were performed using the Promethion Metabolic Cage System (Sable Systems International, Las Vegas, NV, USA) as previously published [20]. Mice were acclimatised for 72 h in the Promethion system before data acquisition began. All Promethion measurements were performed on animals at 13–14 weeks of age.

### Quantitative real-time PCR

Mice were culled between 13.00 and 16.00 h by cervical dislocation and decapitation. Brains were collected and frozen flat on dry ice, then stored at −80 °C. Hypothalamic and brainstem regions were dissected and subsequent RNA extractions were performed using either an RNeasy micro kit (Qiagen) for hypothalamic samples or TRIzol® reagent (Sigma) for the brainstem samples according to the manufacturer’s instructions. In total, 500 ng of total RNA per sample was taken for cDNA synthesis with oligo (dT)20 and random hexamers using the SuperScript III First-Strand Synthesis System for reverse transcription-PCR (Invitrogen). Quantitative real-time PCR reactions were carried out using the Lightcycler 480 Probes Mastermix and Universal Probe Library assays on the Lightcycler 480 System (Roche) with RPL used as an internal standard. For this analysis, seven brains per sex from mice on chow were used, five brains per sex from mice in each HFD group were used and four brains per sex from mice in the diet group were used.

### Statistical analyses

All data are expressed as means ± SEM. No randomisation or blinding was used. The number of mice used was chosen based on our 25 years of expertise in animal research. Data were normally distributed with equal variances between sample groups. Differences between groups were assessed using ANOVA followed by Tukey’s multiple comparison post hoc test, repeated measures ANOVA followed by Bonferroni’s multiple comparison post hoc test, or Student’s *t* test where appropriate and as stated in the figure legends. Statistical analyses were performed with Prism, version 9 (GraphPad Software Inc, La Jolla, CA, USA). *p* = 0.05 was taken to be statistically significant.

## Results

### Female mice are resistant to fat mass gain during the transition to a HFD

**Fig. 1:**
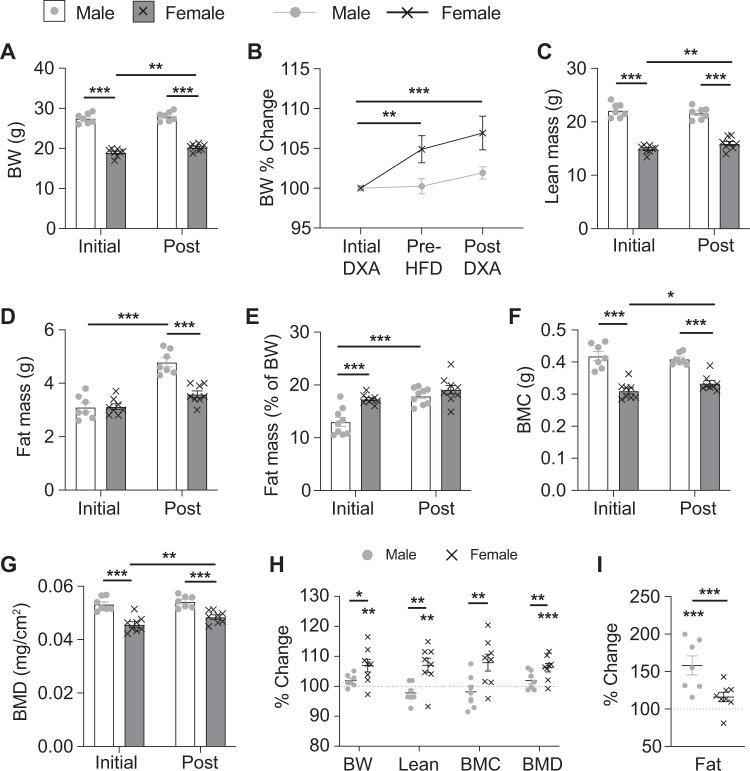
Body composition changes during the transition to a HFD. **A** Absolute and **B** percentage change of body weight before (initial) and after (post) entering the Promethion metabolic cages and the transition to HFD. Pre-HFD timepoint is taken from the Promethion measurements immediately prior to the transition to HFD. **C** Lean mass, **D** absolute fat mass, **E** percentage fat mass adjusted to body weight, **F** bone mineral content (BMC), and **G** bone mineral density (BMD) as measured by DXA analysis before (initial) and after (post) entering the Promethion metabolic cages and the transition to a HFD. **H** Percentage change in body weight (BW), lean mass, BMC, BMD, and **I** fat mass for each individual mouse between the DXA analyses. Data are means ± SEM of eight per group. Data were analysed by RM two-way ANOVA. *p < 0.05, **p < 0.01, ***p < 0.001 as indicated.

Continuous comprehensive metabolic profiling was performed on 13–14 week old C57BL/6J mice for 3 days prior (chow) and 3 days immediately after the transition to a HFD. Body composition data were obtained by DXA prior to acclimatisation in the metabolic cages and immediately after, with a marked difference observed between the sexes in response to HFD. Male mice showed no change in absolute body weight or lean mass, however they did display a significant increase in fat mass (Fig. 1A–E). In contrast, female mice showed a small but significant increase in total body weight due to an increase in lean mass, BMC and BMD (Fig. 1A–G), but no change in fat mass despite the HFD. However, when including body weight measurements from within the Promethion metabolic cages the body weight increase can be seen to occur during the period on chow prior to the commencement of HFD, with no change in body weight during the HFD period (Fig. 1B). This study was designed in order to be able to investigate changes within each individual mouse and thus when analysed as percentage change, female mice displayed significant changes in body weight, lean mass, BMC and BMD but not fat mass while male mice had a significant change in fat mass only (Fig. 1H, I). In fact, males had an average increase in fat mass of 58 ± 13% which was remarkable given the fact they had been fed a HFD for only 4 days.

### Female mice are resistant to the obesifying effects of HFD transition due to metabolic differences not food intake

**Fig. 2:**
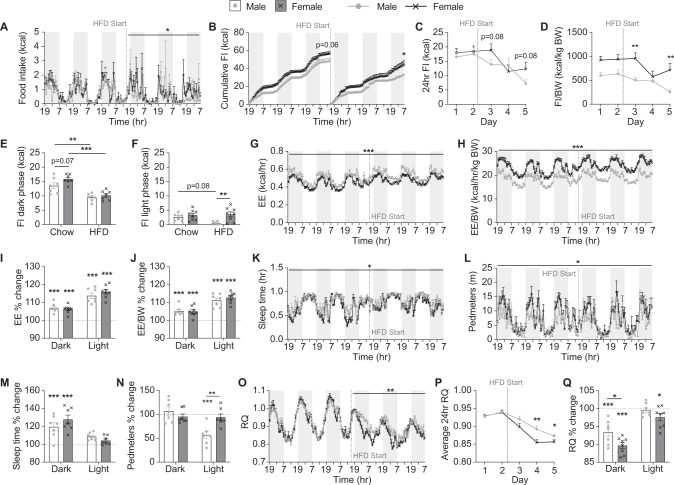
Metabolic changes during the transition to a HFD. **A** Hourly food intake (FI), **B** cumulative hourly FI, **C** 24 h FI, **D** 24 h FI adjusted to body weight, and average daily FI on chow and HFD during the (**E**) dark and (**F**) light phases continuously measured within Promethion metabolic cages. **G** Energy expenditure (EE), **H** EE adjusted to body weight, percentage change in **I** EE and **J** EE adjusted to body weight in the dark and light phases on HFD compared to chow, **K** time spent asleep, **L** distance in voluntary locomotion travelled (pedmeters), percentage change in **M** time spent asleep and **N** pedometers in the dark and light phases on HFD compared to chow, **O** average hourly respiratory quotient (RQ), **P** average 24 h RQ, and **Q** percentage change in RQ in the dark and light phases on HFD compared to chow measured within Promethion metabolic cages. Shaded grey areas represent dark phase, dotted line indicates start of HFD feeding. Data are means ± SEM of eight per group. Data were analysed by RM two-way ANOVA except for **B** where final cumulative differences were assessed by Student’s *t* test. *p < 0.05, **p < 0.01, ***p < 0.001 as indicated.

The lower fat mass gain seen in female compared to male C57BL/6J mice as they transition to a HFD is consistent with previous reports that female C57BL/6J mice are more resistant to the obesifying effects of a HFD [17, 18]. In order to determine whether this is due to a lower caloric intake, we investigated food intake in our mice as they transitioned to a HFD. We found that despite their lower body weight, female mice had a trend towards higher chow intake than male mice, evident primarily during the dark phase (Fig. 2A, B, E, F). However, when switched to HFD, although both sexes reduced their caloric intake, this reduction happened slower and to a lesser extent in female than male mice resulting in female mice showing significantly higher food intake on HFD than males (Fig. 2A–F). Thus, the ability of female mice to resist the obesifying effects of exposure to a HFD does not appear to be due to reduced caloric intake compared to male mice.

Compared to males, female mice had significantly lower absolute energy expenditure levels but when adjusted for body weight or lean mass, their energy expenditure levels were significantly higher (Fig. 2G, H and data not shown). However, there was no difference between the sexes in the response of energy expenditure to the switch to HFD with values significantly increasing to a similar extent in both sexes (Fig. 2I, J). A significant sex difference was also observed in physical activity levels during chow-fed conditions, with females showing less time asleep and an overall increase in pedestrian locomotion (pedmeters), particularly in the dark phase (Fig. 2K, L). After switching to a HFD, both sexes displayed a significant increase in the time spent asleep in the dark phase (Fig. 2K, M). Interestingly however, whilst female mice retained their normal pedmeter levels after the switch to HFD, male mice showed a significant drop during the light phase (Fig. 2L, N). This reduction in activity in male mice could at least partly explain their higher fat mass gain.

Respiratory quotient (RQ) was similar between sexes on chow suggesting similar fuel choice throughout the day and night. However, a marked difference was observed upon the switch to HFD. RQ values dropped in females quicker and to a greater extent than in males, particularly in the dark phase (Fig. 2O, P) suggesting that females have a greater ability to burn fat as a source of fuel. As food intake is reflected in RQ values and the females consumed more food on HFD than males, a lower RQ would be expected. However, the increase in HFD food intake was observed during the light phase (Fig. 2F) whereas the lower RQ values were more pronounced during the dark phase (Fig. 2Q). Therefore, these data suggest that despite consuming more energy than males upon consumption of excess dietary fat, female mice are better able to turn the fat into fuel instead of storage.

### Female mice continue to be more resistant to the obesifying effects of a HFD following sustained exposure

**Fig. 3:**
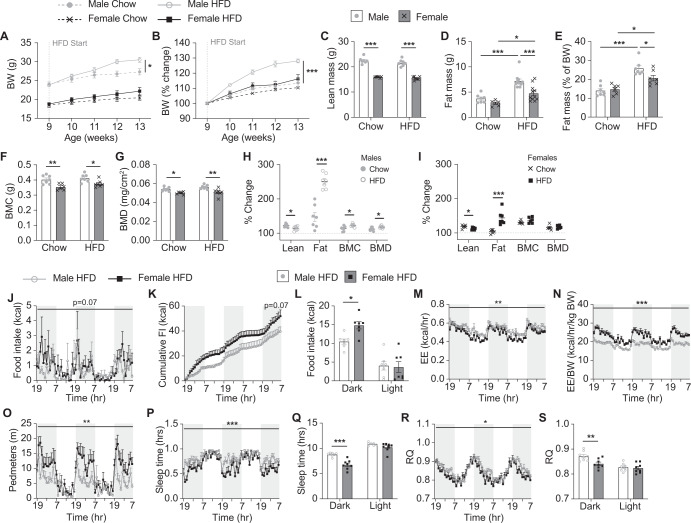
Body composition and metabolic effects following sustained exposure to a HFD. Weekly (**A**) absolute and **B** percentage body weight (BW) and **C** lean mass, **D** absolute fat mass, **E** percentage fat mass adjusted to body weight, **F** bone mineral content (BMC) and **G** bone mineral density (BMD) as measured by DXA on chow- and HFD-fed male and female mice. Percentage change in lean, fat, BMC, and BMD for individual (**H**) male and (**I**) female mice on chow and HFD. **J** Hourly food intake (FI), **K** cumulative hourly FI, **L** average FI during the dark and light phases, **M** energy expenditure (EE), **N** EE adjusted to BW, **O** distance in voluntary locomotion travelled (pedmeters), **P** time spent asleep, **Q** average time spent asleep during the dark and light phases, **R** average hourly respiratory quotient (RQ), and **S** average RQ during the dark and light phases for HFD-fed male and female mice as measured within the Promethion metabolic cages. Shaded grey areas represent dark phase. Data are means ± SEM of 7–8 per group. Data were analysed by **A**, **B** RM two-way ANOVA, **C**–**G** two-way ANOVA, **H**, **I** Student’s *t* test, and **J**–**T** RM two-way ANOVA except for **K** where final cumulative differences were assessed by Student’s *t* test. *p < 0.05, **p < 0.01, ***p < 0.001 as indicated.

In order to investigate whether metabolic responses to the transition to a HFD differ following a more sustained exposure to HFD, we also fed a second group of mice HFD for a period of 5 weeks from 9 weeks of age. An additional group of age-matched chow-fed mice were included as controls for body weight and body composition analysis via DXA. In males, as expected, HFD led to a significant increase in body weight compared to chow-fed mice (Fig. 3A, B). This was due to significantly higher total fat mass but similar absolute lean mass, BMC, and BMD compared to chow-fed males (Fig. 3C–G). Female HFD-fed mice on the other hand had a similar body weight, lean mass, BMC and BMD to chow-fed mice but did display a significantly smaller increase in fat mass (Fig. 3A–G). However, when analysed for percentage change from a pre-HFD DXA for each individual mouse, we found that compared to chow-fed mice, HFD feeding led to a significant decrease in lean mass gain and a significant increase in fat mass in both sexes, although the extent of fat mass gain was markedly higher in males (251%) than in females (143%) (Fig. 3H, I). Interestingly, male but not female HFD-fed mice also showed a significantly greater gain in BMC and BMD than chow-fed mice, likely due to increased weight bearing as a result of their greater body weight (Fig. 3H, I).

After 4 weeks, the HFD-fed mice were comprehensively monitored in the Promethion metabolic cages. Female HFD-fed mice displayed a trend towards increased overall food intake which was significantly higher during the dark phase (Fig. 3J–L). This was significantly higher than that seen in females during the initial transition to HFD (Supplementary Fig. 1A, B) but similar to caloric intake observed in chow-fed females (Fig. 2E, F, comparison not shown). In contrast, male HFD-fed mice continued to display a reduction in caloric intake compared to chow-fed males, similar to that observed in the initial transition to HFD (Supplementary Fig. 1A, B).

Whilst energy expenditure levels in both sexes increased compared to chow upon introduction of HFD, they were not further altered after sustained exposure to HFD (Supplementary Fig. 1C–F). Females continued to display greater physical activity compared to males, which was now significant during the dark phase as well as the light phase (Fig. 3O and Supplementary Fig. 1G, H). However, whilst both sexes spent more time asleep during the dark phase when initially exposed to a HFD (Fig. 2), this was no longer the case in female mice after 4 weeks on HFD creating a significant difference between the sexes (Fig. 3P, Q and Supplementary Fig. 1I, J). The amount of time spent asleep during the dark phase for female mice fed a HFD for 4 weeks was similar to that seen in female chow-fed mice (chow: 6.2 ± 0.3 h; HFD: 6.7 ± 0.3 h; *p* = 0.5) but remained elevated in male HFD-fed mice (chow: 7.3 ± 0.2 h; HFD: 8.7 ± 0.1 h; *p* = 0.004). Therefore, these data further suggest that the higher fat mass gain observed in males may be at least partially due to lower physical activity and increased time spent asleep.

Interestingly, both male and female mice displayed a further drop in RQ during the dark phase after 4 weeks on a HFD compared to that observed in the early transition period (Supplementary Fig. 1K, L). However, females continued to display a significantly lower RQ during the dark phase than males (Fig. 3R, S) suggesting that they maintain their greater ability to utilise fat as a fuel source following sustained exposure to HFD, consistent with their lower body fat gain.

### Male and female mice respond similarly to a return to chow diet after sustained HFD feeding

**Fig. 4:**
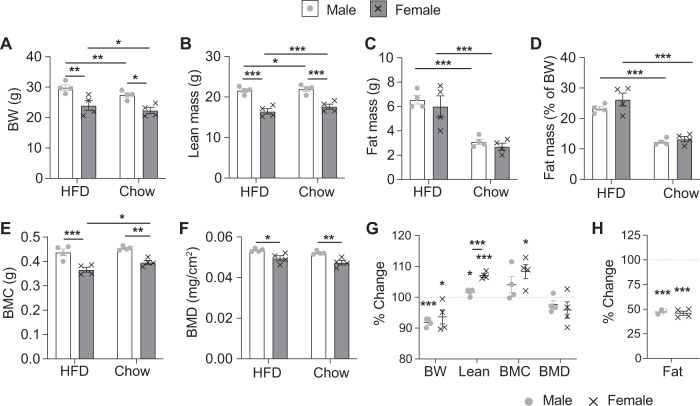
Body composition changes following the return to a chow diet after sustained HFD feeding. **A** Body weight and **B** lean mass, **C** absolute fat mass, **D** percentage fat mass adjusted to body weight, **E** bone mineral content (BMC), and **F** bone mineral density (BMD) as measured by DXA analysis on male and female mice on HFD (4 weeks) and 3 days after transitioning back to chow. **G** Percentage change in body weight (BW), lean mass, BMC, BMD, and **H** fat mass for each individual mouse between the DXA analyses. Data are means ± SEM of four per group. Data were analysed by RM two-way ANOVA. *p < 0.05, **p < 0.01, ***p < 0.001 compared to HFD or as indicated.

The mice that were fed a HFD for 5 weeks were switched back onto a chow diet whilst still in the Promethion metabolic cages to determine whether there were any differences between the sexes in their response to a situation representative of dieting. DXA analyses were performed before and after they entered the Promethion system, with the second analysis performed 72 h after the return to a chow diet. Strikingly, few differences were observed between the sexes with all mice quickly reverting to a state similar to mice who have never been fed a HFD. Both males and females lost significant body weight due to an ~50% reduction in fat mass despite a significant increase in lean mass (Fig. 4A–D, G, H). The increase in lean mass was more pronounced in female than male mice (Fig. 4B, G) who also showed a significant increase in BMC but not BMD (Fig. 4E–G). Remarkably, after only 3 days back on chow, these “diet” mice in fact had similar body weight, lean mass and fat mass values to age and sex matched mice that had been maintained constantly on chow (Supplementary Fig. 2A–D). However, both sexes displayed a significantly higher BMC, but not BMD, to chow-fed controls, likely due to greater weight bearing during their time on HFD (Supplementary Fig. 2E, F).

**Fig. 5:**
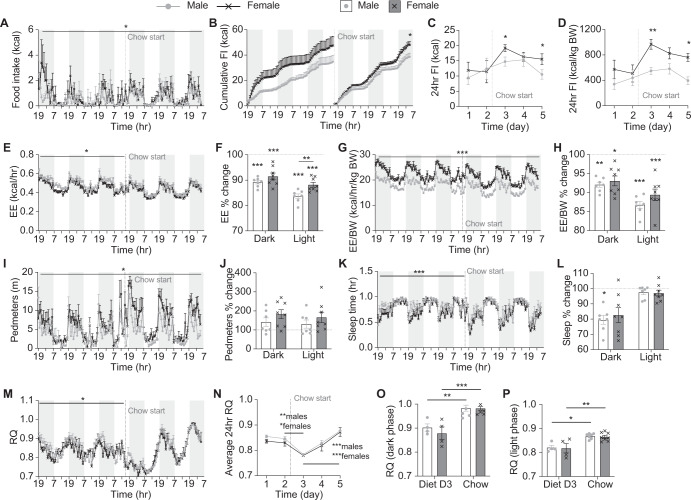
Metabolic changes following the return to a chow diet after sustained HFD feeding. **A** Hourly food intake (FI), **B** cumulative hourly FI, **C** 24 h FI, and **D** 24 h FI adjusted to body weight (BW) continuously measured on male and female mice within Promethion metabolic cages on HFD followed by a switch to chow as indicated. **E** Energy expenditure (EE), **F** percentage change in EE in the dark and light phases on chow compared to when on HFD, **G** EE adjusted to BW, **H** percentage change in EE adjusted to BW in the dark and light phases on chow compared to when on HFD, **I** distance travelled in voluntary locomotion (pedmeters), **J** percentage change in pedmeters in the dark and light phases on chow compared to when on HFD, **K** time spent asleep, **L** percentage change in time spent asleep in the dark and light phases on chow compared to when on HFD, average (**M**) hourly and **N** 24 h respiratory quotient (RQ), and average 24 h RQ in the **O** dark and **P** light phases on day 3 of chow (Diet D3) compared to that of constantly chow-fed mice measured within Promethion metabolic cages. Shaded grey areas represent dark phase, dotted line indicates start of chow feeding. Data are means ± SEM of eight per group. Data were analysed by RM two-way ANOVA except for **B** where final cumulative differences were assessed by Student’s *t* test. *p < 0.05, **p < 0.01, ***p < 0.001 compared to HFD or as indicated.

Overall food intake in males did not immediately alter upon the return to chow with mice consuming a similar caloric intake as they did when on HFD (Fig. 5A–D). However, a clear difference was observed between the sexes, with females consuming more than males upon the return to chow (Fig. 5A–D). Whilst the average 24 h chow intake over these 3 days was similar to mice that had been constantly on chow (Supplementary Fig. 2G, H), on day 3, male but not female mice displayed a significantly reduced food intake compared to the average daily intake of constantly chow-fed male mice (diet males: 10.5 ± 1.5 kcal/24 h; chow males: 17.0 ± 1.7 kcal/24 h; *p* = 0.02).

Upon the return to chow, energy expenditure levels immediately dropped in both sexes back down to levels similar to that of chow-fed mice, although male “diet” mice actually displayed even lower absolute energy expenditure values, and females lower values when normalised to body weight then chow-fed mice (Fig. 5E–H and Supplementary Fig. 2I–L). Locomotor activity did not significantly change in either sex with females remaining more active than males throughout the time in the Promethion (Fig. 5I, J) and males remaining less active than chow-fed mice, at least during the light phase (Supplementary Fig. 2M, N). However, male mice did show a reduction in the amount of time spent asleep during the dark phase (Fig. 5K, L) bringing them back to levels seen in chow-fed mice (Supplementary Fig. 1O, P).

Interestingly, RQ dropped significantly and to a similar extent in both sexes in the first 24 h immediately following the reintroduction of chow, before slowly climbing each day after that (Fig. 5M, N). However, the average RQ values on day 3 after chow reintroduction were still significantly lower than that seen in constantly chow-fed mice for both sexes with no difference between the sexes (Fig. 5O, P). These data suggest that immediately following the switch to chow from a HFD, both male and female mice source a greater proportion of their fuel from their fat reserves. This gradually changes as their fat mass decreases and appears to be de-regulated from food intake.

### Sexually dimorphic food intake responses to HFD may be due to altered NPY signalling in the brainstem

**Fig. 6:**
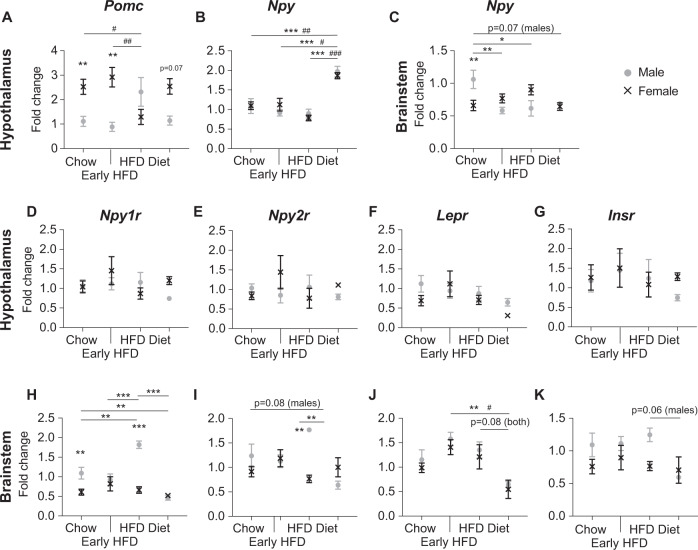
mRNA expression levels of Pomc, Npy and associated receptors in the hypothalamus and brainstem. Quantification of **A** proopiomelanocortin (*Pomc*), and **B** neuropeptide Y (*Npy*) mRNA in the hypothalamus, and **C** *Npy* in the brainstem of male and female mice either maintained on chow (chow), 4 days after a transition to HFD (early HFD), after 5 weeks of HFD feeding (HFD) and 4 days after a transition from sustained HFD to chow (diet). Quantification of **D** *Npy1r*, **E** *Npy2r*, **F** *Insr*, and **G** *Lepr* mRNA in the hypothalamus as well as **H** *Npy1r*, **I** *Npy2r*, **J** *Insr*, and **K** *Lepr* mRNA in the brainstem of male and female mice on the various diets. Data are means ± SEM of at least four per group. Data were analysed by two-way ANOVA. *p < 0.05, **p < 0.01, ***p < 0.001 between sexes or between males of different groups as indicated. ^#^*p* < 0.05, ^##^*p* < 0.01, ^###^*p* < 0.001 between females of different groups as indicated.

The hypothalamus is a key brain site for the integration of multiple circulating signals of hunger and satiety including leptin and insulin which both act in a negative feedback manner to inhibit anabolic (NPY/AgRP neurons) and stimulate catabolic (POMC/CART neurons) effector pathways thus supressing food intake and thereby inhibiting further weight gain [21, 22]. Therefore, we investigated mRNA expression levels of *Pomc* and *Npy* in the hypothalamus between the different sexes and groups. Consistent with previous publications [23, 24], *Pomc* mRNA levels were significantly higher in female than male mice on chow. This was maintained at the early HFD timepoint but significantly reduced to levels similar to male mice during sustained HFD feeding and returned to normal upon the reintroduction of chow (Fig. 6A). In contrast, no difference in *Npy* mRNA levels were observed between the sexes under any conditions or with HFD feeding, however a marked increase was observed under “diet” conditions (Fig. 6B). As *Npy* is also expressed in regions of the brainstem important in the control of energy metabolism [25], we also analysed *Npy* mRNA levels in the brainstem. Interestingly, we observed a significantly lower *Npy* mRNA level in the brainstem of female mice on standard chow diet compared to males (Fig. 6C). Furthermore, *Npy* mRNA levels in male but not female mice significantly decreased following the introduction and sustained intake of HFD and remained decreased upon the reintroduction of chow (Fig. 6C).

We next investigated the mRNA expression of NPY’s Y1 and Y2 receptors (*Npy1r* and *Npy2r*) as well as receptors for leptin (*Lepr*) and insulin (*Insr*). Interestingly, there were no significant differences observed in the hypothalamus for any of these genes either between the sexes on each diet or between diets for each sex (Fig. 6D–G). However, in the brainstem, we observed significantly higher mRNA expression for the *Npy1r* in male mice on chow which further increased under sustained HFD before dropping to levels similar to female mice under “diet” conditions (Fig. 6H). *Npy2r* expression was also elevated in male HFD-fed mice and dropped to levels lower than chow-fed mice under diet conditions (Fig. 6I). *Lepr* mRNA expression in the brainstem was significantly reduced in both sexes under diet conditions compared to HFD-fed mice, with a similar trend observed for *Insr* mRNA expression in male mice only (Fig. 6J, K).

## Discussion

Our systematic approach shows that male and female C57BL/6J mice have markedly different metabolic responses to the introduction as well as the sustained intake of a HFD but surprisingly consistent responses to a dieting situation. Investigating the behavioural, metabolic, and hormonal signalling changes that occur in adult mice as they transition from chow to HFD and back again has uncovered interesting differences between the sexes with important implications for the influence of sex on short-term changes in dietary fat consumption including binge eating and dieting.

Whilst higher activity levels and less time spent asleep likely contribute to the resistance to the obesifying effect of a HFD in female mice, the most marked difference we observed was in RQ levels. The importance of RQ is consistent with a previous publication by Longo et al. showing that locomotor activity did not explain body weight gain in long-term HFD-fed male C57BL/6J mice, but RQ was correlated with energy intake and changes in body mass [19]. In our study, whilst males and females had virtually identical daily RQ fluctuations under standard chow conditions, females showed a significantly greater drop immediately upon the introduction of a HFD, indicative of a greater proportion of their fuel coming from fat, and maintained a lower RQ than males throughout the HFD. In animals with a steady energy balance, fat mass remains constant and average 24 h RQ values are equal to FQ values which are defined by the macro-nutrient composition of a diet. In situations of positive energy balance, RQ values are above FQ values and weight gain results through fat storage [19]. The estimated FQ value for our HFD diet (0.847) is, as expected, lower than that for chow (0.942). The fact that female mice were able to reduce their RQ values quicker than males towards the HFD FQ value indicates that they were able to adapt more quickly to the change in diet and were more capable of maintaining a state of energy balance and thus lower fat mass gain. Remarkably, upon the switch back to chow, the difference in RQ between the sexes immediately ceased. These data suggest that female mice have a greater ability to utilise fat in the diet as a source of fuel, but that both sexes have a similar ability to burn fat reserves for fuel. Further studies will be needed to elucidate the underlying mechanisms driving the greater ability of female mice to utilise fat in the diet as a source of fuel.

The protection against obesity in female mice has been attributed to the effects of circulating oestrogen levels on glucose and insulin homoeostasis, body fat distribution, pro-inflammatory markers, and hepatic lipogenesis [26–29]. Ovariectomy has been shown to eliminate the protection of female mice to gaining weight on a HFD [30]. Thus, age-dependent reductions in oestrogen may be a factor in the eventual development of obesity in some long-term HFD feeding studies. However, here we show that female C57BL/6 mice had higher hypothalamic *Pomc* and lower brainstem *Npy* mRNA expression levels than male mice under standard chow conditions. The higher *Pomc* expression is consistent with previous reports [23, 24] and suggests that inherent differences in the strength of catabolic versus anabolic neurological signalling pathways between the sexes which may play a role in the response to HFD feeding. The higher *Npy* mRNA levels in the brainstem of male mice reduced upon the introduction of a HFD whereas the higher *Pomc* mRNA levels in female mice were more resistant to change only dropping after sustained exposure to a HFD. The reduced *Npy* levels in the brainstem of male mice on a HFD may explain the sustained reduction in caloric intake observed in male but not female mice. Interestingly, it has been shown that not only do female mice have more *Pomc* neurons but their *Pomc* neurons also display higher neural activity. Furthermore, the deletion of the transcription factor TAp63 in *Pomc* neurons decreased their activity and increased the susceptibility to HFD-induced obesity in female mice, but the same deletion did not affect male mice [24]. Taken together, these data suggest that elevated *Pomc* neuronal activity in C57BL/6J mice is important in the sexual dimorphism observed in response to HFD although this may wane with long-term exposure.

In contrast to the sexually dimorphic responses seen with increased dietary fat, it was striking to observe virtually no differences between the sexes in their response to a dieting situation. Interestingly, the dramatic change in body composition in the “diet” mice was associated with reduced *Lepr* expression in the brainstem and significantly elevated hypothalamic *Npy* mRNA levels. Leptin is primarily secreted from adipose tissue in direct proportion to fat mass and acts in an inhibitory manner on Npy neurons to prevent further increases in energy intake [31]. Thus, these data suggest that the marked reduction in fat mass observed upon the reintroduction of chow after a period of HFD led to reduced leptin signalling, removing the inhibition on hypothalamic Npy neurons and resulting in situation reminiscent of a starving brain. Further studies would be required to determine the long-term effects of this response and its contribution to the often-ineffective nature of short-term diets in humans.

Previous studies looking at the effect of sex on diet-induced obesity have shown a similar resistance to the obesifying effects of long-term HFD feeding in female C57BL/6J mice [17, 18, 26]. A recent paper by Casamiro et al., compared metabolic parameters in male and female mice after either 8 or 16 weeks of two differing high-fat diets [17]. Of note, in this paper, they began the diets in young, 4-week-old mice whereas we have investigated the effect of a diet change in adult mice. The strength of our study is that we have investigated the change in metabolic parameters upon diet change within the same mice whilst they remained in the metabolic cages. In contrast, Casamiro et al. have investigated metabolic parameters in different groups of mice which, combined with the young age at which they started the diet interventions, could account for subtle differences between the studies.

Several previous studies including recent genetic diversity screening panels have shown that mouse strains can differ substantially in their metabolic response to HFD feeding, as indeed is also the case with other phenotypes as well [32–34]. However, these studies have predominantly been performed on male mice only and often do not address sex-specific effects. Whilst the results presented here may be specific to the C57BL/6J strain, which was chosen as it is the most commonly used reference strain in diabetes and obesity research [35], they highlight the need to include both sexes in future metabolic studies. Further work to elucidate the underlying peripheral mechanisms driving these metabolic differences between males and females will allow for the development of better preventative and treatment strategies for people suffering from weight issues and associated complications.

## Supplementary information

Supplementary Figures

## Data Availability

The data generated and analysed during the current study are available from the corresponding author on reasonable request.
