# Supplementary material for: Sex-specific changes in metabolism during the transition from chow to high-fat diet feeding are abolished in response to dieting in C57BL/6J mice
Source: Int J Obes (Lond). 2022 Jul 6;46(10):1749–58. doi: 10.1038/s41366-022-01174-4 (PMC9492540; doi:10.1038/s41366-022-01174-4)
Supplement: Supplementary file 1 — Supplementary Figures [file 41366_2022_1174_MOESM1_ESM.pdf]

Supplementary Figure Legends

Supplementary Figure 1: Average food intake in the (A) dark and (B) light phases, average energy expenditure (EE) in the (C) dark and (D) light phases, average EE normalised to body weight (BW) in the (E) dark and (F) light phases, average pedmeters travelled in the (G) dark and (H) light phases, average time spent asleep in the (I) dark and (J) light phases, and average respiratory quotient (RQ) in the (K) dark and (L) light phases in male and female mice in the first 3 days of a transition to a HFD (Early) compared to after 4-5 weeks of HFD feeding. Data are means ± SEM of at least 7 per group. Data were analysed by two-way ANOVA. * = $p < 0.05$, ** = $p < 0.01$, *** = $p < 0.001$ as indicated.

Supplementary Figure 2: (A) Body weight (BW), (B) lean mass, (C) fat mass, (D) fat mass as a percentage of BW, (E) bone mineral content (BMC), and (F) bone mineral density (BMD) in male and female mice 4 days after transition back to chow following 4 weeks of HFD feeding (diet) compared to age matched controls maintained on chow diet (chow). Average food intake in the (G) dark and (H) light phases, average energy expenditure (EE) in the (I) dark and (J) light phases, average EE normalised to BW in the (K) dark and (L) light phases, average pedmeters travelled in the (M) dark and (N) light phases, average time spent asleep in the (O) dark and (P) light phases, and average respiratory quotient (RQ) in the (Q) dark and (R) light phases in male and female mice under diet conditions compared to chow-fed controls. Data are means ± SEM of at least 4 per group. Data were analysed by two-way ANOVA. * = $p < 0.05$, ** = $p < 0.01$, *** = $p < 0.001$ as indicated.

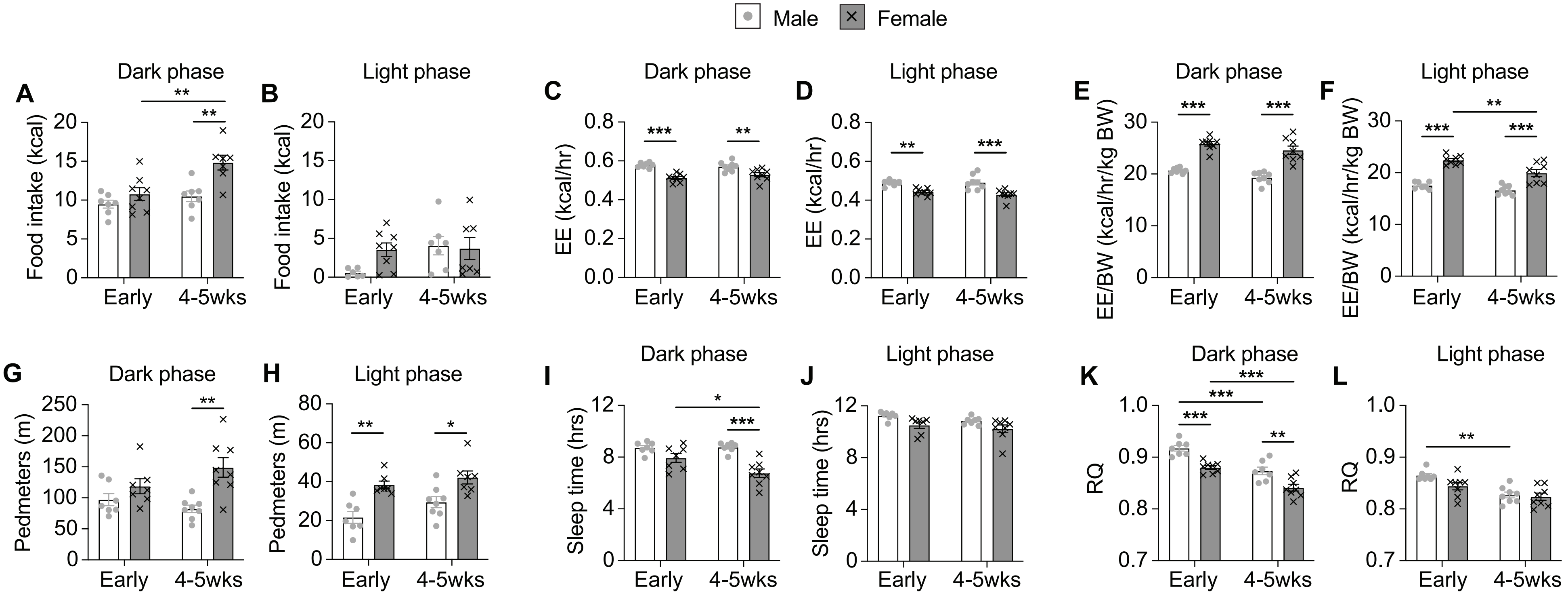

Supplementary Figure 1

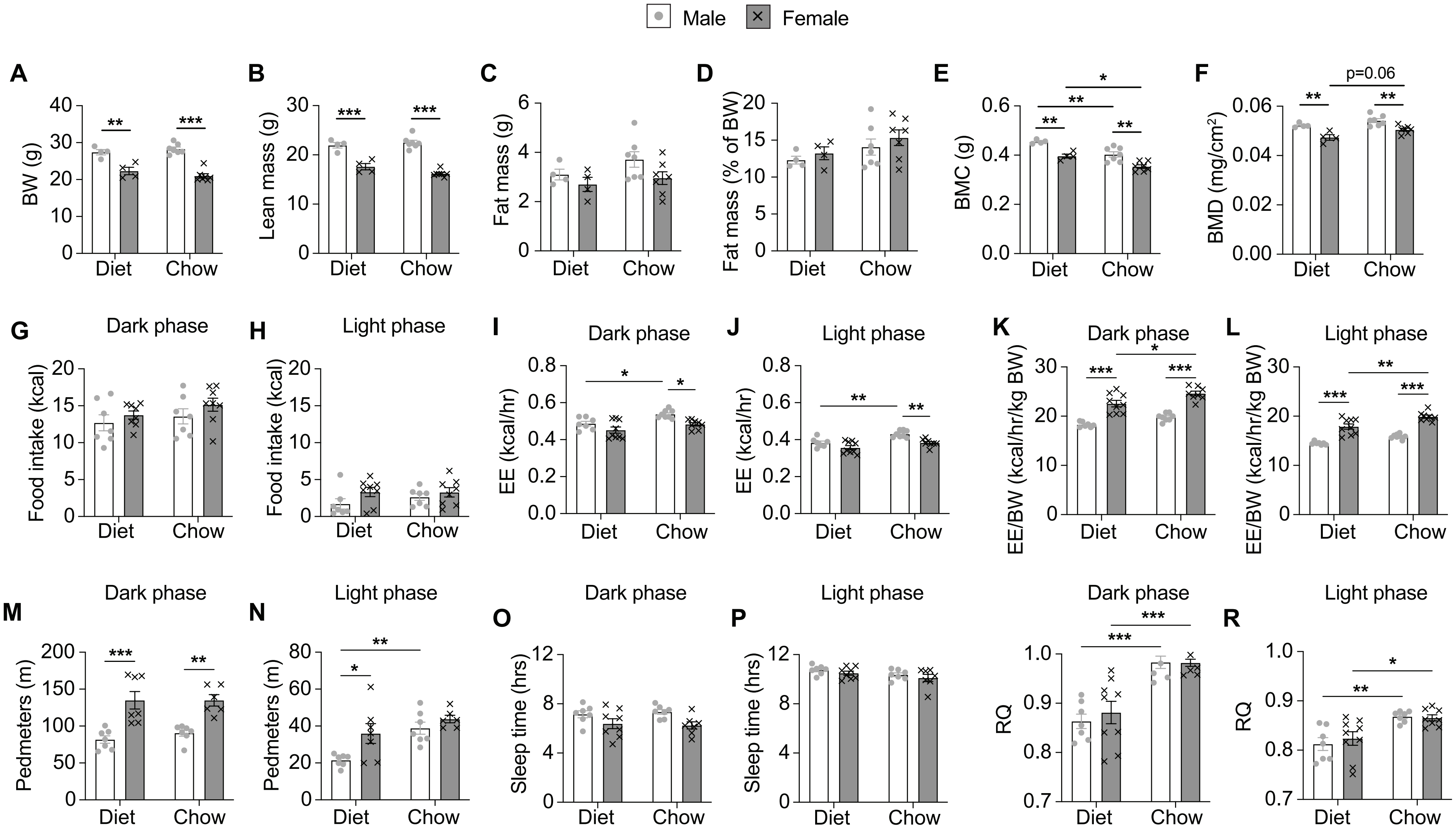

Supplementary Figure 2
